# Tapered Quantum Cascade Laser Arrays Integrated with Talbot Cavities

**DOI:** 10.1186/s11671-018-2617-z

**Published:** 2018-07-09

**Authors:** Yue Zhao, Jin-Chuan Zhang, Feng-Min Cheng, Dong-Bo Wang, Chuan-Wei Liu, Ning Zhuo, Shen-Qiang Zhai, Li-Jun Wang, Jun-Qi Liu, Shu-Man Liu, Feng-Qi Liu, Zhan-Guo Wang

**Affiliations:** 1 Key Laboratory of Semiconductor Materials Science, Institute of Semiconductors, Chinese Academy of Sciences, Beijing, 100083 China; 2 Beijing Key Laboratory of Low Dimensional Semiconductor Materials and Devices, Beijing, 100083 China; 3 College of Materials Science and Opto-Electronic Technology, University of Chinese Academy of Sciences, Beijing, 101408 China

**Keywords:** Phase locked arrays, Quantum cascade lasers, Semiconductor lasers

## Abstract

Power scaling in broad area quantum cascade laser (QCL) usually leads to the deterioration of the beam quality with an emission of multiple lobes far-field pattern. In this letter, we demonstrate a tapered QCL array integrated with Talbot cavity at one side of the array. Fundamental supermode operation is achieved in the arrays with taper straight-end connected to the Talbot cavity. Lateral far-field of the fundamental supermode shows a near diffraction limited beam divergence of 2.7^°^. The output power of a five-element array is about three times as high as a single-ridge laser with an emission wavelength of around 4.8 μm. However, arrays with the taper-end connected to the Talbot cavity always show a high-order supermode operation whatever Talbot cavity length is.

## Background

Quantum cascade laser (QCL), invented in 1994, has been one of the most important light sources in mid- and far-infrared for its wavelength flexibility and portability [1–3]. Popular applications of QCLs have covered many areas such as free space optical communication and directed infrared countermeasure (DRICM), trace chemical sensing of explosives, toxins, pollutants, and medical testing [4–7]. Some applications always demand high output light power for better jamming effect and detection accuracy. High power QCLs can be obtained by broadening the width of active region area. However, a simple broadening of the ridge without waveguide engineering design or external optics will deteriorate the beam quality of QCLs with an emission of multiple lobes far-field pattern [8]. Single-lobe emission is obtained in the past with the methods such as photonic crystal distributed-feedback (PCDFB) QCLs, angled cavity QCLs, master-oscillator power-amplifier QCLs, and broad area QCLs via external feedback mechanisms [9–12]. Recently, phase-locked arrays have been popular approaches to keep wide ridge QCL emitting with coherent narrow beam patterns.

Phase-locked arrays have been skillfully applied in the wide ridge and low divergence semiconductor lasers since the 1980s [13]. In previous works, phase-locked QCL arrays have been studied in the Y-junction arrays, resonant leaky-wave-coupled arrays, and evanescent wave-coupled arrays, as the near-infrared laser did in the past [14–18]. These structures either bring about large losses in the waveguide [15] or result in heat accumulation by pursuing a short adjacent distance to obtain the coupling [16–18]. Recently, diffraction-coupled QCL arrays that integrated a side cavity based on diffraction-coupled Talbot effects were reported [19]. In the diffraction-coupled structure, the coupling occurs in the Talbot cavity by the diffraction of the ridge end and reflection of the cavity facet. The diffraction-coupled phase-locked QCL array elements can be placed for a wide space, which will decrease the heat accumulation.

**Fig. 1:**
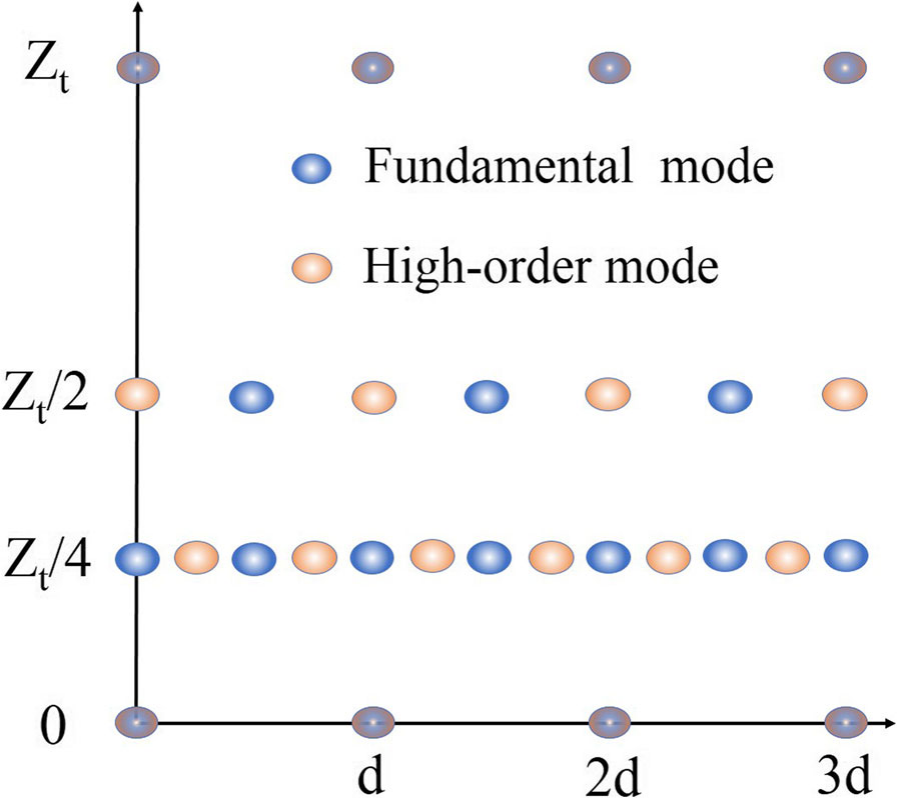
Fundamental and high-order supermode distribution at fractional Talbot planes. Blue ovals correspond to fundamental supermodes, and brown ovals correspond to high-order supermodes

Talbot effect is a well-known optical phenomenon that a periodic structure can produce self-images at certain regular distances [20]. This effect has been exploited to phase-locked lasers in the near-infrared, which is called diffraction coupling scheme phase-locked array [21–23]. In this method, a flat mirror should be placed in front of the cavity facet of the laser array to provide optical feedback. The length between the mirror and the array facet is so-called Talbot distance, which is defined as

$$ {Z}_t=\frac{2n{d}^2}{\lambda } $$

where *n* is the refractive index of the material, *d* is the center-to-center distance of the array, and *λ* is the free space wavelength. The supermodes which are reflected into the array channels will obtain the self-reproductive oscillation. Figure 1 shows the distribution of the fundamental supermode and high-order supermode in a fractional Talbot distance. Once the supermodes in the *Z*_*t*_/4 position are reflected into the array channels, fundamental supermode superposition and operation will be extracted.

The output power of Talbot cavity phase-locked QCL arrays is limited because of a low coupled efficiency between the Talbot cavity and the array channels. To further increase the output power of the Talbot cavity QCL arrays, the filling factor (ratio of ridge width to period) should be increased. Whereas, widening the channel width will arise a high-order mode emission of the array elements. Reducing the center-to-center distance will increase the heat accumulation. Taper structure is one of the best methods to increase the filling factor at the same time ensuring a fundamental mode operation of the single ridge itself. In this letter, taper structures are exploited and the Talbot cavities are integrated at one side of taper structures respectively. The devices with straight-end connected to Talbot cavity show a fundamental supermode operation with a near diffraction limited (D.L.) far-field divergence of 2.7°. In the contrast, the devices with taper-end connected to Talbot cavity show high-order supermode operation whatever Talbot cavity length is. A maximum peak power of 1.3 W is obtained for the devices with straight-end connected to Talbot cavity with a threshold current density of 3.7 kA/cm^2^ and a slope efficiency of 0.6 W/A at 298 K.

## Methods

The QCL wafer was grown on an n-doped (Si, 2 × 10^17^ cm^−3^) InP substrate wafer by solid-source molecular beam epitaxy (MBE). The active region (AR) structure consists of 35 periods of strain-compensated In_0.67_Ga_0.33_As/In_0.37_Al_0.63_As quantum wells and barriers. The whole wafer structure before the fabrication is 4 μm lower InP cladding layer (Si, 3 × 10^16^ cm^−3^), 0.3-μm-thick n-In_0.53_Ga_0.47_As layer (Si, 4 × 10^16^ cm^−3^), 35 active/injector stages, 0.3-μm-thick n-In_0.53_Ga_0.47_As layer (Si, 4 × 10^16^ cm^−3^), 2.6-μm InP upper cladding layer (Si, 3 × 10^16^ cm^−3^), 0.15-μm InP gradually doped layer (changing from 1 × 10^17^ to 3 × 10^17^ cm^−3^), and 0.4-μm highly doped InP cladding layer (Si, 5 × 10^18^ cm^−3^).

**Fig. 2:**
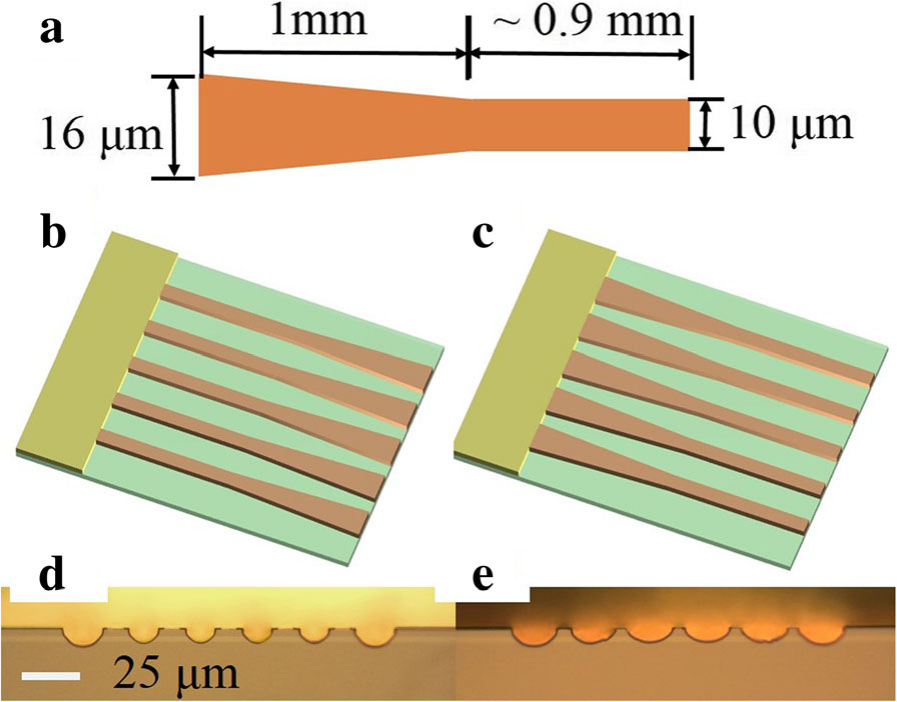
**a** Sketch of the taper element in the arrays; the 3D schematic of the arrays with **b** straight-end connected to Talbot cavity and **c** taper-end connected to Talbot cavity, corresponding to the front facet microscope images of **d** and **e**

After the epitaxy in MBE, the devices were etched with the wet chemical etching method and then deposited 450 nm SiO_2_ with plasma-enhanced chemical vapor deposition (PECVD). After opening the electrical injection window, the top metal contact was formed. The two sections of the Talbot cavity and Tapered array are electrically connected through the Au top contact. Then, the wafer substrate was thinned and the bottom contact metal contacts were evaporated. The wafer was cleaved into around 2 mm long with a dicing saw to precisely control the Talbot cavity length. Finally, the devices were soldered epilayer side down onto the copper heat sink with indium solder. Since the Talbot cavity section is electrically injected, the heat will accumulate for its wide dimension, which should be avoided by employing the electrical insulation in the future work. The Talbot cavity section probably can be replaced with other waveguide material by employing the complicated fabrication such as wafer bonding and alignment, and the phase-locked operation may still be achieved. According to the supermode distribution of the Talbot cavity in Fig. 1, our Talbot cavity length was determined to be *Z*_*t*_/8 similar to ref. [19] which is around 104 μm in this letter. Figure 2 shows the sketch and microscope pictures of the device. The arrays contain five taper elements and a Talbot cavity. The taper element consists of a 1-mm-long taper-end and ~ 0.9-mm-long straight-end with a width changing from 10 to 16 μm. The center-to-center spacing between adjacent elements in the array is 25 μm, and the length of each laser device is around 2 mm. The length of Talbot cavity in this paper is all around 104 μm.

## Results and Discussion

**Fig. 3:**
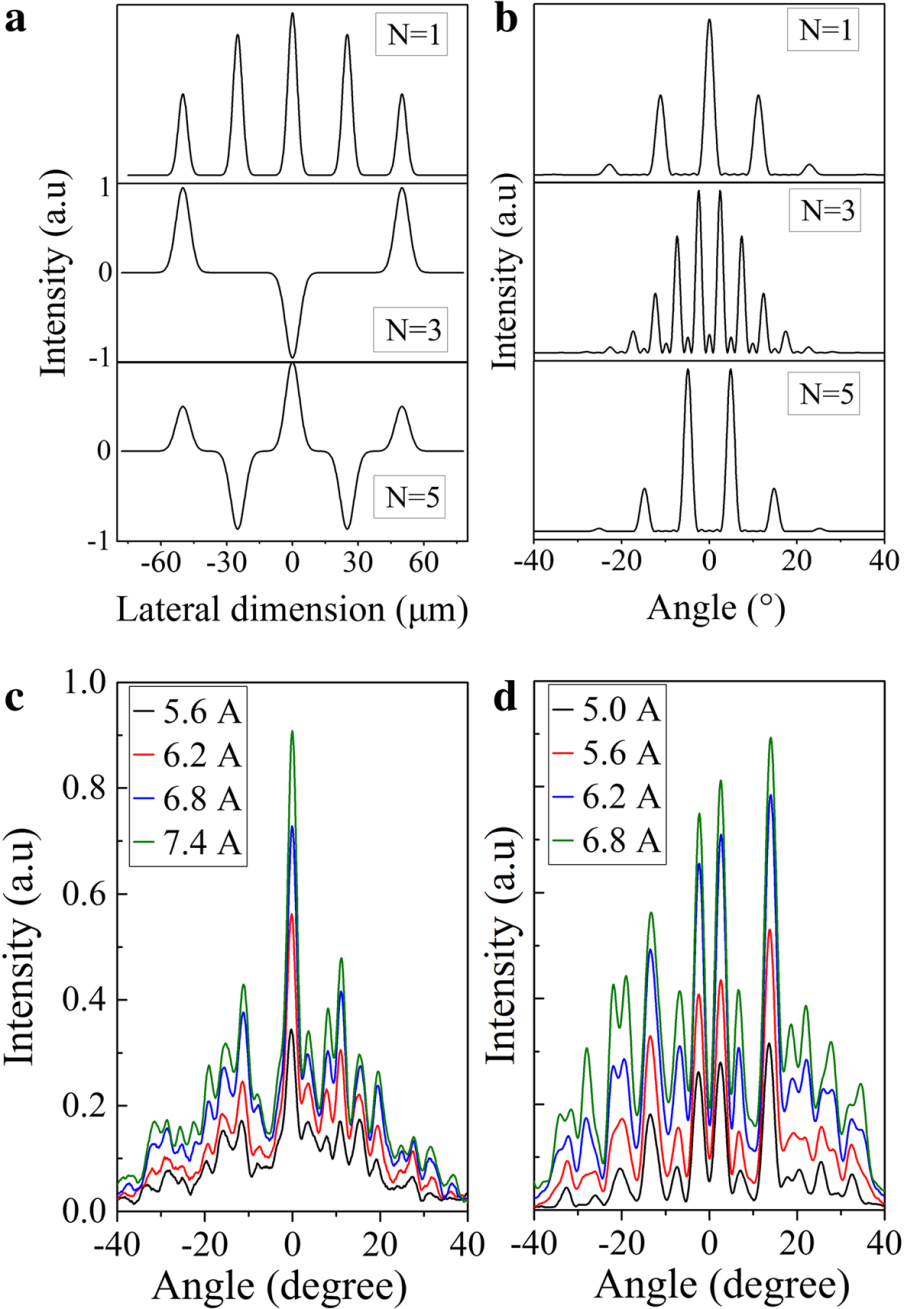
**a** Calculated near-field patterns of the *N* = 1, 3, 5 order supermodes in a five-element diffraction-coupled array. The fundamental supermode (*N* = 1) is calculated based on the straight-end connected to the Talbot cavity, and the high-order supermodes (*N* = 3, 5) are based on taper connected to the Talbot cavity. **b** The simulated far-field patterns according to **a**. **c** The measured far-field distribution of QCL array with straight-end connected to a Talbot cavity. **d** The measured far-field distribution of QCL array with taper-end connected to a Talbot cavity

According to the coupled mode theory, the number of supermodes in phase-locked array is same as the number of elements [24]. For instance, phase-locked array with five elements will have the five supermodes. Assuming only the adjacent coupling between the array elements in the Talbot cavity, the near-field distribution pattern of the different order supermode can be obtained with the coupled matrix [24]. The near-field strength changing as a function of the array lateral dimension can be demonstrated as [25]:

$$ {E}_j\propto \sum \limits_{m=1}^M\sin \left(\frac{mj}{M+1}\pi \right)\exp \left[-\frac{{\left(x-{x}_m\right)}^2}{\omega^2}\right] $$

where *j* is the order of supermode, *M* is the numbers of array elements, *ω* is the waist of the Gauss beam in each element, and *x*_*m*_ is the central location of each element. The simulation results of different order supermodes are shown in Fig. 3a. The corresponding far-field patterns can be deduced with the Fourier transform from the near-field distribution, as shown in Fig. 3b.

The far-field patterns of the Talbot cavity phase-locked arrays were measured from the array waveguide facet using the lock-in technique with a room temperature mercury-cadmium-telluride (MCT) detector. The QCL array mounted on a rotation stage was placed ~ 25 cm away from the MCT detector and controlled by a home-built software for data collection. The measured far-field patterns of Talbot cavity arrays are shown in Fig. 3c, d, corresponding to the straight-end connected to the Talbot cavity device and the taper-end connected to the Talbot cavity device. The far-field distributions in Fig. 3c show strong central lobes at 0°, indicating the existence of fundamental supermode operation according to couple-mode theory. The full width of half maximum (FWHM) is around 2.7°, which shows a diffraction-limited (D.L.) divergence angle according to the D.L. formula: sin *θ* = 1.22*λ*/*d*, where *θ* is the D.L. angle, *λ* is wavelength, and *d* is the light output width of the array. For a tapered single emitter with a light output width of 16 μm, the D.L. FWHM divergence is around 21°. The side-lobes appear around ~ 12° which are very close to the FWHM location of the single emitter far-field envelope. The intensities of the central lobe and side-lobes are corresponding to the distribution of single emitter far-field pattern. Thus, the side-lobes have the half of intensity of the central lobe. Furthermore, single-lobe far-field profile array can be obtained by increasing ridge width to decrease the divergence of array elements. The wider ridge width can be achieved by widening the taper. The far-field patterns in Fig. 3d have no lobe at center 0° position, but are primarily double-lobed, showing the operation of higher order supermodes, which are corresponding to the three-order supermode in Fig. 3b. In order to obtain the fundamental supermode operation, we fabricated the devices with the different Talbot cavity length from 90 to 110 μm stepping 1 μm. Unfortunately, the fundamental supermode operation in the device with taper-end connected to the Talbot cavity cannot be obtained whatever Talbot cavity length is.

**Fig. 4:**
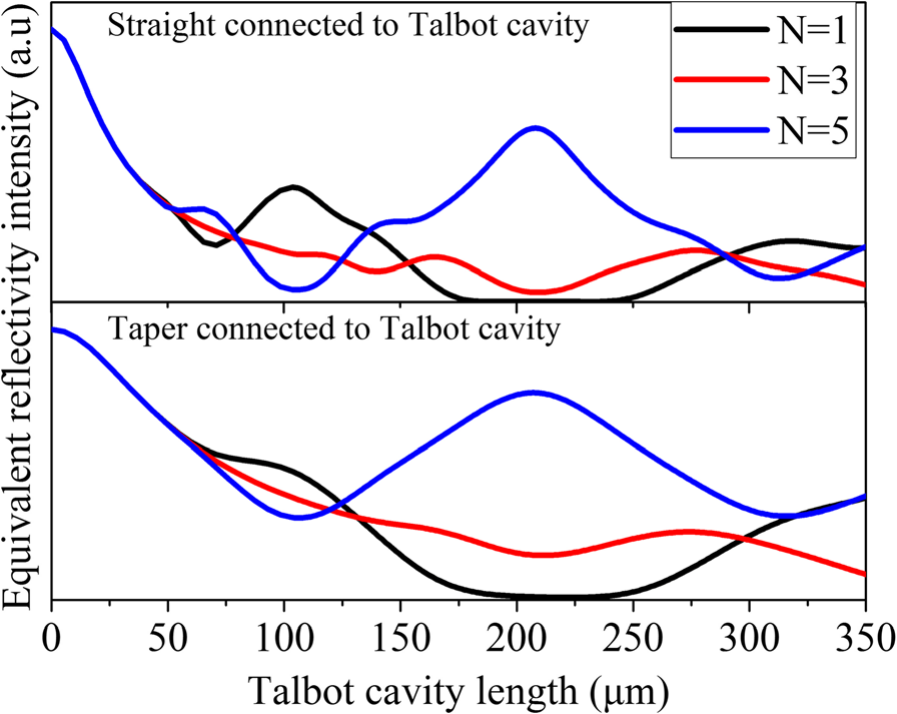
Theoretical equivalent reflectivity intensity of the Talbot cavity changes as a function of the Talbot cavity length for *N* = 1, 3, 5 order supermodes of a five-element Talbot cavity QCL array, the upper shows the straight-end connected to the Talbot cavity and the lower shows the taper-end connected to Talbot cavity

The far-field results of two type arrays can be explained with the theoretical model in ref. [19, 21]. The Talbot cavity can be approximated as a reflect mirror with different equivalent reflectivity for different supermodes; the high equivalent reflectivity means a high gain efficiency and a low threshold gain. The calculation and simulation of the equivalent reflectivity are similar to the ref. [19]. Figure 4 shows the simulation results of equivalent reflectivity for different order supermodes changing as a function of the Talbot cavity length. Since the *N* = 2, 4 order supermodes in phase-locked arrays always have larger waveguide loss than *N* = 1, 3, 5 order supermodes, they are neglected in the simulation here. For the straight-end connected to Talbot cavity arrays, the fundamental supermode has the highest equivalent reflectivity and large discrimination compared with the high-order supermodes around *Z*_*t*_/8. For the taper-end connected to the Talbot cavity, the discrimination between the fundamental supermode and high-order supermode is relatively small. In this case, the laser tends to work with three-order supermodes due to weak mode discrimination in the taper-end connected to the Talbot cavity device.

**Fig. 5:**
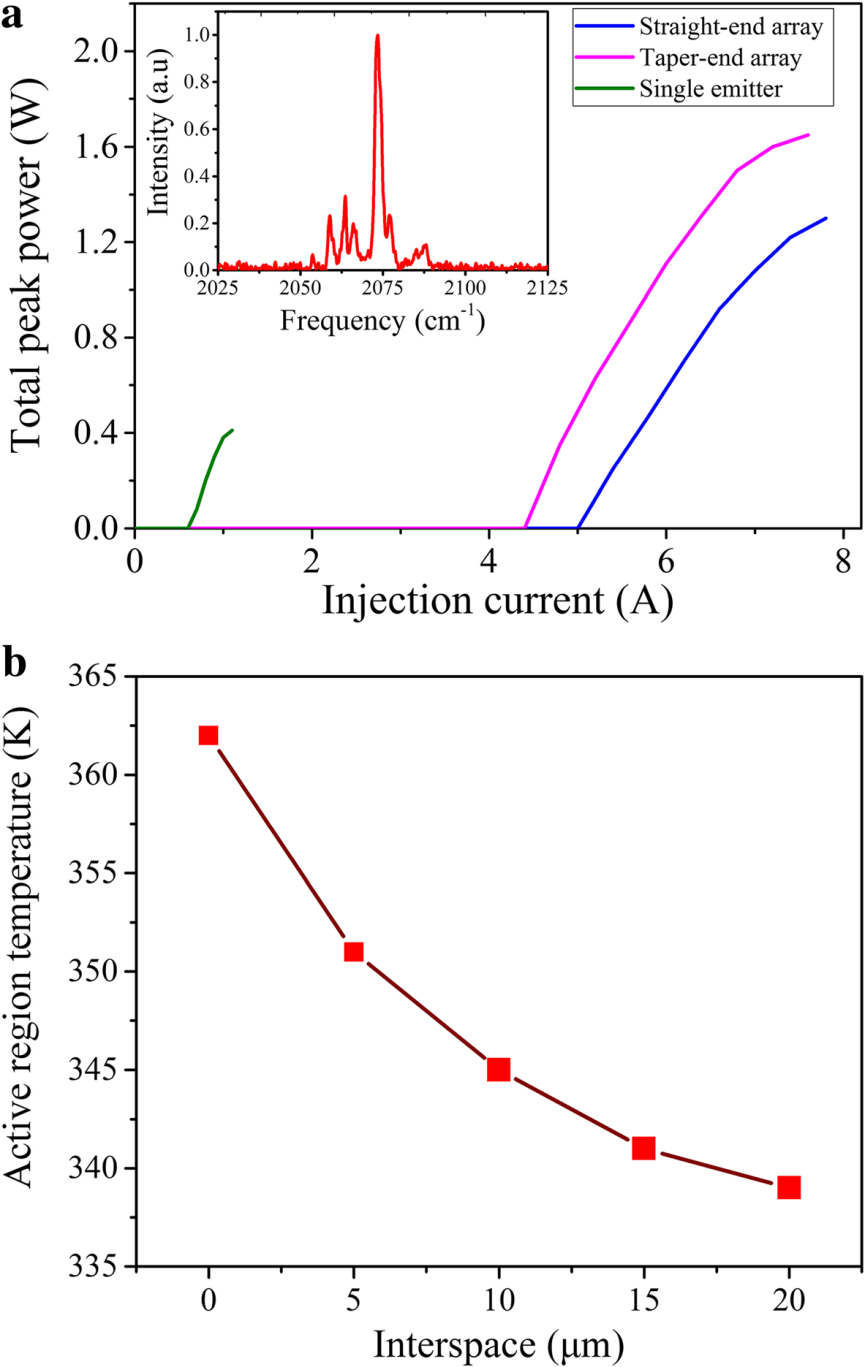
**a** Total peak power change as a function of the injection current at 298 K for straight-end (blue line)/taper-end (purple line) connected to the Talbot cavity QCL array and a 2-mm-long × 10-μm-wide single laser (green line). All of the devices have no coating on both sides of the cavity. The current driver is maintained at 2 kHz with a duty circle of 0.2%. The inset is the lasing spectrum of the straight-end arrays at 1.3 times threshold current, which peaked around 4.8 μm. **b** The active region temperature of QCL array changing as a function of array elements interspace. The array elements ridge width is fixed as 10 μm, and the interspace changes from 0 to 20 μm with a step of 5 μm

The emitted optical power was measured with a calibrated thermopile detector placed directly in front of the laser waveguide facet. The spectrum measurements were performed using a Fourier transform infrared (FTIR) spectrometer with 0.25 cm^−1^ resolution in rapid scan mode. Figure 5a shows the power-current (P-I) characteristic under pulsed mode with current driver maintained at 2 kHz with a duty circle of 0.2%. For the device with straight-end connected to the Talbot cavity QCL array, a total peak power of 1.3 W is obtained at 298 K with a threshold current density of 3.7 kA/cm^2^ and a slope efficiency of 0.6 W/A, corresponding to 1.6 W output power with a threshold current density of 3.4 kA/cm^2^ and a slope efficiency of 0.65 W/A for the taper-end array as shown in blue line and purple line. As a contrast, the single laser device with 2-mm-long × 10-μm-wide ridge shows a maximum peak power of 0.41 W, a threshold current density of 3 kA/cm^2^, and a slope efficiency of 1 W/A. The output power of the arrays with the fundamental operation is three times of single emitter. To more briefly present the tested results, the output characteristic of three devices are summarized in Table 1. The average output power from each element is about 63% of the single emitter, which is higher than that in ref. [19]. Ref. [26] reports a phase-locked QCL array with an intra-cavity Talbot filter with the average power of individual array element equal to 43% of a single emitter. The efficiency is lower than the devices with one junction between the Talbot cavity and the array elements because of additional optical loss in the two circular junction caused by the wet etching method. Ref. [27] reports a six-element device integrated with a Talbot cavity with five times output power of single emitter with a coupling efficiency about 83%. The lower efficiency in our devices is most likely due to stronger edge diffraction losses in the Talbot cavity and the fabrication with wet etching method. The following work should adopt the dry etching method and increase the taper zone length to obtain further power scaling. The inset of Fig. 4a shows the lasing spectrum of the phase-locked arrays at room temperature and 1.3 *I*_th_. The center wavelength was measured to be 4.8 μm with a multi-mode nature resulting from the lack of a longitudinal-mode selection mechanism. The single-mode spectrum can be achieved by introducing a distributed feedback (DFB) grating on the top cladding layer. The thermal characteristic of broad QCLs and QCL arrays are simulated with the finite element software COMSOL. The fixed ridge width is set as 10 μm and the interspace of the array elements changing from 0 to 20 μm at a step of 5 μm. Figure 5b shows the temperature of AR changing as a function of the element interspace. The temperature of AR in the wide ridge device is about 20 K higher than that in the Talbot cavity device.

**Table 1:** Output Characteristic of the Three Different Devices

	Straight-End	Taper-End	Single
Total Peak Power (W)	1.3	1.6	0.41
Threshold Current Density (kA/cm^2^)	3.7	3.4	3
Slope Efficiency (W/A)	0.6	0.65	1
Relative power (a.u)	3.25	4	1

## Conclusion

In conclusion, we have demonstrated the tapered QCL arrays integrated with Talbot cavities in straight-end and taper-end respectively. The devices with the Talbot cavity integrated at the straight-end shows a fundamental mode far-field patterns with a D.L. divergence of 2.7° at an emission wavelength of 4.8 μm. An output power of 1.3 W is obtained for the straight-end array with a slope efficiency of 0.6 W/A. Since the Talbot cavity phase-locked array does not require a very close coupling distance, the heat accumulation is lower than the evanescent wave-coupled arrays. Such devices have a potential for high brightness QCL arrays of high duty cycle operation with D.L. divergence. Future work should focus on the selection of an appropriate array element ridge width and interspace, the use of buried ridge waveguides, and the thermal management with micro-impingement coolers [28]. In addition, the reduced cascade number of the AR will make great contribution to the high duty cycle operation of high brightness QCLs [29].

## Abbreviations

AR: Active region
CW: Continuous wave
D.L.: Diffraction-limited
DFB: Distributed feedback
FWHM: Full width of half maximum
*I*_th_: Threshold current
MBE: Molecular beam epitaxy
MCT: Mercury-cadmium-telluride
MOVPE: Metal organic vapor phase epitaxy
PECVD: Plasma-enhanced chemical vapor deposition
P-I: Power-current
QCL: Quantum cascade laser
WPE: Wall-plug efficiency
